# Preliminary slide scanner throughput evaluation in a intensive digitization facility setting

**DOI:** 10.1186/1746-1596-8-S1-S45

**Published:** 2013-09-30

**Authors:** Vincenzo Della Mea, Giampiero Duglio, Filippo Crivelli, Pierluigi Banfi, Giancarlo Chiovini

**Affiliations:** 1 Dept. of Mathematics and Computer Science, University of Udine, Italy; 2 Cloud Pathology Group, Milano, Italy; 3 Department of Pathology, Azienda Ospedaliera Ospedale di Circolo di Busto Arsizio, Busto Arsizio, Italy

## Background

Whole Slide Imaging (WSI), called also Digital Microscopy, is the most current approach to digitization of histological information [1,2]. It allows for transferring a whole histological slide into digital form, thus enabling any kind of digital treatment from storage and transmission, to telediagnosis, to automatic image analysis. WSI technologies developed only recently, and thus most uses described in literature are coming from research and teaching applications [3]. However, one acknowledged potential use of this technology is also aimed at dematerializing slide archives, by bringing them in digital form inside a so-called PACS (Picture Archiving and Communication System) [4]. This application would provide a major boost in the adoption of WSI in the routine work of a clinical pathology laboratory.

Two main differences can be recognised between academic applications like research and teaching, and routine application in the pathology laboratory: slide volume, and diagnostic reliability.

The former difference is related to the number of involved glass slides and the time span on which scanning occurs. Teaching in particular, but also research applications, foresee the acquisition of a limited number of slides, in terms of either total number or scanning needs per time unit. In fact, teaching with digital slides usually involves slides accumulating into a teaching archive that may slowly grow, in years and years, but with no massive amounts of slides involved. Research trials instead might involve the scanning of a large number of slides but in a limited time frame, related to the life span of the research project, and that can be archived offline at the end of the project.

Both cases differ from the routine acquisition in a clinical pathology laboratory, where there is the need for a sustained acquisition of a fraction (or all) the glass slides daily produced by the laboratory, to be made available to pathologists when needed. This means that the scanning procedure should be as efficient as possible, and in particular able to perform a sustained scanning as quick as glass slide production is in the specific laboratory. This means also that there is need for personnel (e.g., laboratory technicians) that feed the scanner with glass slides, start the scanning procedure, check associated patient data, verify results, unload slides, etc. Any technical hitch occuring in those phases (e.g., software bugs, slide loading difficulties, etc) is likely to decrease the overall thoughput of the scanner.

The latter difference is related to how digital slides are used. Scanning is not a process without errors: loss of information is always present, and derives in part from the process itself, in part from specific features and pitfalls of the scanning device, in part from the preparation technical quality of the source glass slide. In teaching applications, slides are selected for their educational meaning, so there is a selection of the acquired material, that allows for recognising scanning errors or simply missing information. Such selection is also made possible by the low number of slides acquired at each scanning session. The same can be considered true for any research usage, since it is done as part of the research project. On the other side, routine scanning is aimed at providing slides for the diagnostic work of the pathologist, either for primary diagnosis or for giving access to previous slides of the same patient when diagnosing a new histologic exam. Thus, diagnostic reliability of the digital slide should be guaranteed, and this means that acquired slides should be as good as possible as they come out from the scanner. Since every device may fail in acquisition of some slide, the least they fail, the better is.

The present paper aims at evaluating the first of the two issues, namely throughput, by means of a trial carried out by submitting to three different slide scanners a large number of glass slides coming from six different Pathology laboratories, to obtain a continuous one-month scanning session. A preliminary evaluation of the second issue, namely diagnostic reliability, has been carried out on a subset of the slides. The experimentation has been carried out to provide a technical basis for the selection of a number of scanners to be applied in a newcoming company that provides outsourced scanning and storage services. Research questions include:

- Do scanners support continous acquisitions on 24/7, in terms of software and hardware behaviour?

- Are manual operations related to scanners repeatable and hassle-free?

- How many slides per day can be truly acquired, taking into account all the operations needed?

- How many slides need to be acquired again due to quality issues?

- Is there any connection between clinical pathology laboratories procedures and glass quality, that influence acquisition quality or speed?

- Is digital diagnosis equivalent to microscope-based diagnosis?

## Material and methods

### Slide scanners

Three scanners (A, B, C) have been provided by three manufacturers by means of their national distributors, which also provided training and technical support during the experimentation. Among the different models in the companies listings, the scanners were chosen among those aimed at high throughput, i.e., with slide loaders ablle to host hundreds of slides.

### Cases and slides

Glass slides have been provided by six Pathology laboratories from Italian hospitals. Glass slides were aimed at representing the average production of those labs, so they have been chosen consecutively from lab archives among biopsies and surgical samples. 1200 slides per lab have been requested.

### Data analysis

The following variables have been recorded for each acquisition:

○ acquisition speed

○ acquisition success

○ barcode acquisition success

○ digital slide size

○ diagnosis

Some more information has been collected regarding the scanning sessions:

○ scanner downtime during experimentation

○ accidental events (slide jams, etc)

For these variables, average, minimum, maximum, totals have been calculated depending on the variable, with data aggregated by scanner, by hospital and by both.

For a first preliminary evaluation of diagnostic performance, about 10% of cases will be randomnly extracted from the whole set and examined by 3 pathologist for each case and for each scanner. This way, every case will receive a total of 9 diagnoses to be compared with the gold standard microscope diagnosis. For comparison, a senior pathologist (FC) will examine all diagnoses and categorize differences on a 0-3 scale (diagnosis not possible; wrong diagnosis; incomplete or inaccurate diagnosis; correct diagnosis).

## Results and discussion

### Glass slides

Each hospital provided at least 1200 glass slides as requested. However, the first scanned batch resulted to have 1244 slides, which have been scanned without a preliminary counting, done thereafter for the other hospitals. Thus, the total amount of scanned slides is 7244.

Cases were representative of the lab production. The case set was composed by an average on 75% biopsies (range: 61%-88%) versus surgical samples; each case included an average of 3.65 slides (range:2.67-5.04), for an average total of 354 cases per lab (range: 238-449). Table 1 shows details.

**Table 1:** Case set features

Lab	slides	cases	%biopsies	slides/case
1	1200	449	77.95%	2,67
2	1200	238	78,57%	5.04
3	1200	250	60,79%	4.80
4	1200	n.a.	n.a.	n.a.
5	1200	433	88.22%	2.77
6	1244	402	69.15%	2.99

	7244	354	74.94%	3.65

### Scanner throughput

Table 2 shows details on scanner throughput.

**Table 2:** Scanner throughput details

Scanner	slides	% success	total time	time/slide	barcode failure %	downtime (h)
A	7332	99,19%	72:47	3:46	1,86%	1,54
B	7200	95,65%	100:38	5:14	1,19%	2,04
C	7210	98,31%	92:43	4:25	0,00%	1,00

	21742	97,72%	89:27	4:28	1,02%	1,53

Scanning succeeded on slightly less than 98% of acquisitions (range: 95.65%-99.19%), with an average time of 4’28” per slide (range: 3’46”-5’14”), higher than values declared by manufacturers. Barcode acquisition failed on a very low number of slides (1%), with most errors in the very initial phases of the experimentation, due to software problems quickly solved by manufacturers programmers. This might reveal that barcode acquisition until now has not been as usual as supposed to be.

All scanners experienced some downtime, averaging at about one hour and half on the whole experimentation, one month long.

When aggregated by laboratory, the same data show a partially unexpected variability, as shown in table 3.

**Table 3:** Acquisition success

Lab	total slides	% success	time/slide	barcode failure %	% biopsies
1	3600	97,86%	5:23	0,55%	77.95%
2	3600	98,25%	4:52	1,06%	78,57%
3	3610	97,34%	3:27	0,69%	60,79%
4	3600	98,44%	3:37	0,85%	n.a.
5	3600	97,03%	4:52	3,47%	88.22%
6	3732	97,39%	4:37	7,14%	69.15%

	21742	97,72%	4:28	1,02%	74.94%

In particular, acquisition time per slide, averaged on the three scanners, ranges from 3’27” to 5’23” – a higher variability than the one we obtained by aggregating per scanner. While a reason could be a different percentage of biopsies versus surgical samples, at first glance this does not seem to explain all the difference, and thus needs further investigation. A candidate reason seems to be the quality of histological preparation, which might not be influencing human vision, but indeed might influence automatic scanning.

### Slide size

A total of about 10 Terabytes have been acquired during the experimentation, which, due to the total number of slides, corresponds roughly to the output of an Italian surgical pathology laboratory (histology only). The experimentation took about 1600 hours of scanning in one month.

Table 4 shows a summary of slide sizes by scanner and by laboratory. Even here a partially unexpected variability is apparent.

**Table 4:** slide sizes

Avg. Slide Size			(GB)		
	A	B	C	***avg***	***% biopsies***
1	0,56	0,52	0,70	0,59	*77*,*95%*
2	0,50	0,53	0,89	0,64	*78*,*57%*
3	0,33	0,30	0,52	0,38	*60*,*79%*
4	0,32	0,30	0,48	0,36	
5	0,36	0,29	0,43	0,36	*88*,*22%*
6	0,41	0,35	0,53	0,43	*69*,*15%*

*avg*	0,41	0,38	0,59	0,46	

### Diagnostic performance

The preliminary diagnostic performance evaluation has been carried out on 116 cases, equivalent to 8.89% of the total number of cases. Each case has been acquired with 3 scanners, so the total number of digital cases has been 348. Cases were attributed at random to pathologists, chosen in order to have each case reviewed by one pathologist from the originating hospital and two pathologists from other hospitals.

Digital diagnosis has been categorized as follows: (0) diagnosis not possible; (1) wrong diagnosis; (2) incomplete or inaccurate diagnosis; (3) correct diagnosis. Table 5 shows details.

**Table 5:** diagnostic agreement

	Scanner A	Scanner B	Scanner C	TOTAL
**No. of cases**	116	116	116	348

**Avg. Agreement level**	**2.655**	**2.681**	**2.310**	**2.548**

**Level 3**	81%	83%	65%	76%

**Level 2**	9%	8%	14%	10%

**Level 1**	5%	4%	9%	6%

**Level 0**	5%	5%	12%	7%

Results, though preliminary and in need of further investigation, seems substantially in line with other similar experimentations [5,6].

## Conclusions

The present study provides novel insights on current slide scanners from the point of view of their massive application in a slide scanning service. As far as we know, no other study attempted yet the same kind of intensive evaluation, though relevant evaluations have been done in the European Scanner Contest series [7]. The present paper illustrates some preliminary findings on scanner throughput and reliability.

Real world scanning time seems higher than declared by manufacturers. However, it seems also dependent on slide preparation quality, thus suggesting that preparing for scanning is more crucial than preparing for microscope and human eye. From this point of view, when in need of routine, massive digital slide scanning, preparation guidelines or standards should be provided for a better and quicker overall operation.

Scanner reliability has been proven to be high and scanning success too, but both are not 100%, so, even if scanning is automatic, it is not possible to do it in a non supervisioned way. This means that personnel should take care of all the steps: loading, scanning, trouble management, informatics and networks issues, etc. From this experience, we can tell that expertise needed is both in laboratory techniques and Information technology. Figures with both expertises are rare, in particular in Italian surgical pathology laboratories.

A peculiar issue is the slide trays role: the least trays are manipulated, the least errors, glass breakings, misidentifications, and time lost. This means that in an ideal situation, they could be used not only to load the scanner , but also as a transport medium and/or as a definitive glass slide storage medium too. Furthermore, their mechanics should be reliable in intensive scanning setups, and misplacement free. The trays of the examined scanners were not equal from this point of view, and suggested that maybe they should be reengineered from the point of view of the user, in this case an outsourced service provider willing to scan hundreds of thousands slides per year.

Acquisition time and slide size varied not only per scanner but also per laboratory: further investigation is needed to obtain better knowledge on this phenomenon, though it seems related to histological preparation quality. From this point of view, guidelines should be provided for a preparation more adequate for scanning.

As a final remark, the infrastructure needed for Digital Pathology is not just a scanner on a spare table in corner of the lab, like most often until now. This approach aimed at the enthusiast pathologist –that scans a limited number of slides, not in a hurry- seems to have guided some design choice by scanner manufacturers, including trays design and partially incomplete barcode scanning software. Routine massive scanning of slides needs solid infrastructure and personnel able to deal with a number of interdisciplinary issues like:

○ backup, power supply, network sizing and management,

○ memory sizing, resizing, and management,

○ software management and upgrade;

○ slide loading, verification, unloading, archival.

At present, it is not clear who may take care of all of this in the current surgical pathology laboratory.

The step beyond is to redesign some features having in mind industrial usage of scanners, in a regular workflow, with standards-based processes, including imaging standards [8-10].

## Competing interests

VDM has received research funding from CloudPathology. The remaining authors declare that they have no competing interests.

## Authors' contributions

GD and VDM designed the study. GD, PB and GC supervised the overall experimentation. FC did the diagnostic performance evaluation. GD, VDM, FC analysed data. VDM wrote the first draft of the paper. All Authors reviewed and modified the paper.
